# Mating Behaviour and Vibratory Signalling in Non-Hearing Cave Crickets Reflect Primitive Communication of Ensifera

**DOI:** 10.1371/journal.pone.0047646

**Published:** 2012-10-19

**Authors:** Nataša Stritih, Andrej Čokl

**Affiliations:** National Institute of Biology, Department of Entomology, Ljubljana, Slovenia; Stanford University, United States of America

## Abstract

In Ensifera, the lack of well-supported phylogeny and the focus on acoustic communication of the terminal taxa hinders understanding of the evolutionary history of their signalling behaviour and the related sensory structures. For Rhaphidophoridae, the most relic of ensiferans following morphology-based phylogenies, the signalling modes are still unknown. Together with a detailed description of their mating process, we provide evidence on vibratory signalling for the sympatric European species *Troglophilus neglectus* and *T. cavicola*. Despite their temporal shift in reproduction, the species’ behaviours differ significantly. Signalling by abdominal vibration constitutes an obligatory part of courtship in *T. neglectu*s, while it is absent in *T. cavicola.* Whole-body vibration is expressed after copulation in both species. While courtship signalling appears to stimulate females for mating, the function of post-copulation signals remains unclear. Mating and signalling of both species were found to take place in most cases on bark, and less frequently on other available substrates, like moss and rock. The signals’ frequency spectra were substrate dependent, but with the dominant peak always expressed below 120 Hz. On rock, the intensity of *T. neglectus* courtship signals was below the species’ physiological detection range, presumably constraining the evolution of such signalling in caves. The species’ behavioural divergence appears to reflect their divergent mating habitats, in and outside caves. We propose that short-range tremulation signalling in courtship, such as is expressed by *T. neglectus*, represents the primitive mode and context of mechanical signalling in Ensifera. The absence of high-frequency components in the signals may be related to the absence of the crista acoustica homologue (CAH) in the vibratory tibial organ of Rhaphidophoridae. This indirectly supports the hypothesis proposing that the CAH, as an evolutionary precursor of the ear, evolved in Ensifera along the (more) complex vibratory communication, also associated with signals of higher carrier frequency.

## Introduction

In Ensifera, the behavioural mechanisms for pair formation are best known for the terminal taxa, including crickets (Gryllidae) and katydids (Tettigoniidae) [1], [2], [3], [4]. These species produce sound signals by friction of forewings (tegminal stridulation) during mating, agonistic and territorial interactions. Ensifera also produce sound by femuro-abdominal stridulation, which occurs in the largely apterous groups. In the non-hearing raspy crickets (Gryllacrididae), Jerusalem crickets (Stenopelmatidae) and splay-footed crickets (Schizodactylidae) this mechanism serves defence [5], [6], [7], while it is also used for social communication in weta (Anostostomatidae), which possess primitive hearing (with tuning that does not match their signals) [8], [9].

Raspy and Jerusalem crickets communicate by vibratory signals produced by body striking (drumming) against the substrate [6], [7]. Drumming and body vibrating without contact with the substrate (tremulation), are performed also by crickets and katydids at close communication distances [10], [11], [12], [13], [14], [15]. This diversity of mechanisms and the behavioural contexts of mechanical signalling in the extant Ensifera impedes our understanding of the evolutionary transition of their signalling modes. Knowledge of this pattern is also incomplete, since communication modes are unknown for two non-hearing, but phylogenetically potentially relevant, ensiferan families − splay-footed crickets and cave crickets (Rhaphidophoridae).

A complex chordotonal organ in the leg tibiae is the main sensory organ for sound and/or vibration detection in Ensifera [16], [17], [18]. The auditory tympanal organ is developed in the fore-legs, as a functional and evolutionary extension of the vibratory tibial organ [19], [20]. Since the ensiferan phylogeny is unresolved [21], it remains unclear as to whether their hearing and sound signalling evolved once, with several subsequent reductions [22], or twice, independently in the cricket and katydid lineages, leaving a part of Ensifera primitively deaf [23]. Further, the exact behavioural context that accompanied the evolution of hearing in Ensifera is still a matter of discussion [23], [24], [25].

Following morphological data and the cladistic approach, Rhaphidophoridae are considered the basal of the primitively non-hearing ensiferan lineages [23], [26]. Support for this hypothesis has been provided by the comparative neuroanatomy of the vibration-sensitive tibial organs and their underlying neuronal network in the non-hearing taxa, strongly suggesting these sensory elements as precursors for audition [25], [27], [28], [29]. Based on these results, Rhaphidophoridae are most relevant for research on the presumably ancestral modes of ensiferan communication.

Rhaphidophoridae are Ensifera, distributed world-wide and regarded as relicts of the Mesozoic tropical forest fauna [30]. Most appear in caves of the temperate zones, while others are nocturnal epigean species [31], [32]. While the family has been generally considered to be mute (e.g. [4]), drumming was reported for some sand-treader and arboreal species [33], [34], and the presence of femuro-abdominal stridulation has been mentioned for the genus *Troglophilus* in some old general literature [35], [36]. In *Troglophilus* species, however, there are clearly no structures that could be involved in stridulation (our own observations). Also, the descriptions of mating from various Rhaphidophoridae have provided no evidence for either sound or vibratory signals [30], [34], [37], [38], [39], [40], [41], [42]. Nevertheless, some mode of vibratory signalling may have been overlooked, since no equipment for recording vibrations was used in these studies.

*Troglophilus neglectus* and *T. cavicola* are relatively closely related Balkan species of the genus distributed in the east Mediterranean [43]. In Slovenia they appear syntopically in most of the investigated caves [44]. Like most other cave-dwelling Rhaphidophoridae, they use caves for daily shelters and leave them at night for foraging, and overwinter in caves [45]. The life cycles span two years in both species but are temporally shifted, so that *T. cavicola* mate between February and April [45] and *T. neglectus* between July and September [46]. During winter diapause they form mixed colonies in caves, though with some differences in the local distribution, indicating their divergent temperature and humidity preferences [45], [47], [48]. At least some *T. cavicola* also mate in caves before leaving them after overwintering [42]. In summer, both species seek for daily shelters close to cave entrances, in litter, under stones, decayed bark, trunks, etc. [43], [45], and *T. cavicola* also climb trees [49]. The males have two pairs of scent glands on the abdomen, which protrude outside the body in the mating season [50]. Apart from this, a brief description of *T. cavicola* mating encountered in a cave [42] is the only information available on the mating behaviour of these species.

The aim of this study was to provide a detailed, quantitative description of the mating process for *T. neglectus* and *T. cavicola*, focusing on the production of substrate-borne vibratory signals. We provide the first recorded document on vibratory signalling for Rhaphidophoridae and demonstrate large behavioural differences between the investigated species. The study contributes substantially to our understanding of the mechanosensory evolution in Ensifera.

## Results

### Pre-mating Behaviour

The main differences observed in pre-mating behaviour of the two species were in protrusion of male abdominal scent glands, the presence of aggressive behaviour and in the general degree of the male activity. These aspects will be summarized here, and described in detail in a further publication.

Males of *T. neglectus* protruded their abdominal scent glands in different behavioural contexts, including excited locomotion associated with either tracking and courting females or male-male aggressiveness. Gland protrusion, male-male antagonism, and excited locomotion were not observed in *T. cavicola*. The animals spent most of the time in the bark overhang, in the hollows in the moss or adjacent to stones, and mating of *T. cavicola* often took place between members of a pair already positioned close to one another for a longer period of time. Although some pairs of *T. neglectus* also showed similar pre-mating behaviour, their copulation was in most cases preceded by the male walking round the terrarium, interacting with the other male and/or courting females.

### The Mating Process

**Figure 1:**
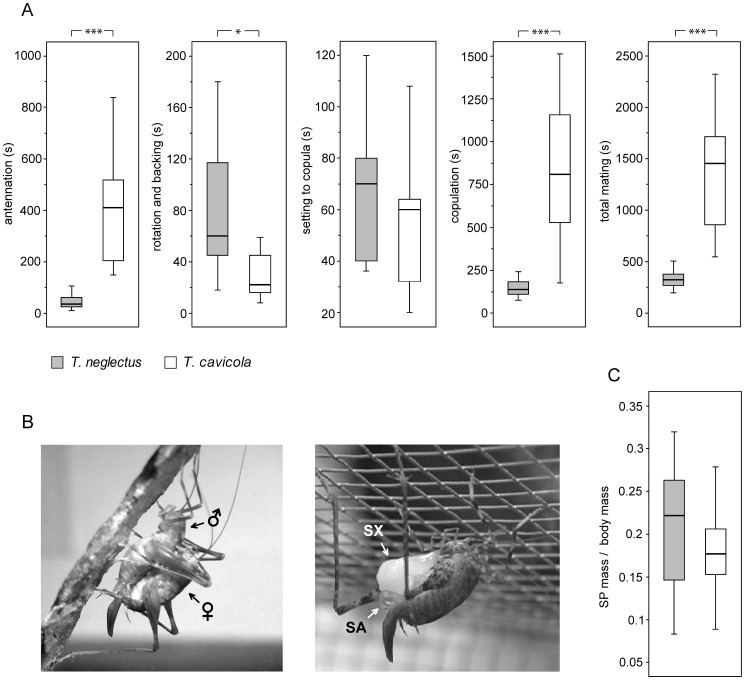
The mating process. A. Duration of successive mating phases and total mating time of *Troglophilus neglectus* and *T. cavicola* (box-whiskers plot with median and interquartile ranges, outliers excluded; the legend is the same for A and C). The asterisks indicate the degree of significance in T-test (* *P*<0.05, *** *P*<0.001). T-test values: antennation *t*_12.305_ = −6.425, *P*<0.001; rotation and backing *t*_28_ = 2.115, *P* = 0.043; setting to copula *t*_28_ = 1.395, *P* = 0.174; copulation *t*_19.317_ = −6.816, *P*<0.001, total mating *t*_13.367_ = −6.180, *P*<0.001. B. A photograph of *T. cavicola* pair in copula (left) and of a female *T. neglectus* feeding on a spermatophore (right; SA − sperm ampula, SX − spermatophylax). C. The spermatophore (SP) mass as a proportion of the body mass compared between the species (T-test values: *t*_21_ = 0.936, *P* = 0.360).

The complete mating process was recorded in 17 of 18 matings that took place in *T. neglectus* and in 13 of 16 matings in *T. cavicola.* In both species mating was most frequently conducted in the bark overhang (see Figure 1B, left), 11 times in *T. neglectus*, 12 times in *T. cavicola* and less often on its upper surface (three times in *T. neglectus,* twice in *T. cavicola*). Both species also mated on moss (twice in *T. neglectus*), stone (once each in *T. neglectus* and *T. cavicola*) and the net cover of the terrarium (twice in *T. neglectus*, once in *T. cavicola*).

In both species the mating process started with antennation, which was followed by male rotation and backing towards the female, establishing the copula and copulation (Figure 1A). In *T. cavicola* duration of the complete process was significantly greater than in *T. neglectus* (for test values see legend for Figure 1). In the former species, its median duration was around 25 minutes (1452 s, Q1−Q3 831−1750.5 s), while in the latter the whole process lasted around 5 minutes (median 312 s, Q1−Q3 246.25−378.75 s). Most of this difference is the result of much longer antennation (median 410 s, Q1−Q3 201.5−520.5 s) and copulation phases in *T. cavicola* (median 810 s, Q1−Q3 501−1200 s) than in *T. neglectus* (median 35 s, Q1−Q3 25−60 s/median 137.5 s, Q1−Q3 107.5−194.5 s).

Qualitative differences between the species were expressed in the first two mating phases. In *T. cavicola*, antennation consisted of a high frequency mutual antennal touching of the partners, while in *T. neglectus* the males were mostly antennating the rather passive females. During rotation and backing towards the female, *T. neglectus* males rhythmically oscillated the abdomen (slightly vibrating the whole body); this was observed visually in all courting males (see also below). After the partners came into contact, these movements resulted in rhythmical stroking of the female’s body. No such movements were observed, and no mechanical signals were recorded, during rotation and backing in *T. cavicola*, where this phase was about three times shorter (median 22 s, Q1−Q3 13−52 s; n = 13) than in *T. neglectus* (median 60 s, Q1−Q3 40−118 s; n = 17; Figure 1A). Antennation followed by abdominal vibration in *T. neglectus* may be referred to as courtship behaviour, since it took place in the same form with both responsive and unresponsive females (and in a few cases also as unspecific courting among the males).

In *T. neglectus*, a median of three successive courtship bouts (Q1−Q3 2−5; n = 17) was needed to induce the female’s response, while in *T. cavicola* the first attempt was successful in most cases (Q1−Q3 1−2; n = 13). With responsive females in *T. neglectus*, by far the longest courtship, composed of tenfold repeated antennation and abdominal vibration, was conducted on the stone, while up to six repetitions of such sequences were needed before copulation was established on the other substrates.

The phase of establishing copula was of similar duration in the two species (Figure 1A). It started with the female climbing on the male’s back, while the male continued backing and grasping over her with lifted hind legs. In copula of both species, the female’s mid-legs were typically clutched between the femur and the tibia of the male’s flexed hind legs (Figure 1B, left). In both species the actual genital coupling took place just shortly before the completion of copula, which was associated with male extrusion of the spermatophore (SP) containing a large spermatophylax (Figure1B, right). The SP had a similar mass in the two species (Figure 1C) and the median value of the proportion of the male’s total body mass was 22.2% in *T. neglectus* (Q1−Q3 13.4−26.8%; n = 12) and 17.7% in *T. cavicola* (Q1−Q3 11.6 − 20.6%; n = 11; for stat. evaluation see legend for Figure 1). In *T. cavicola* the SP extrusion was unsuccessful in three matings, including the males that have been treated with CO_2_ for weighing. This caused a partial extrusion and apparently a change in the structure of the sperm ampula (see Figure 1B, right), which then could not be grasped by the female’s genital structures.

**Figure 2:**
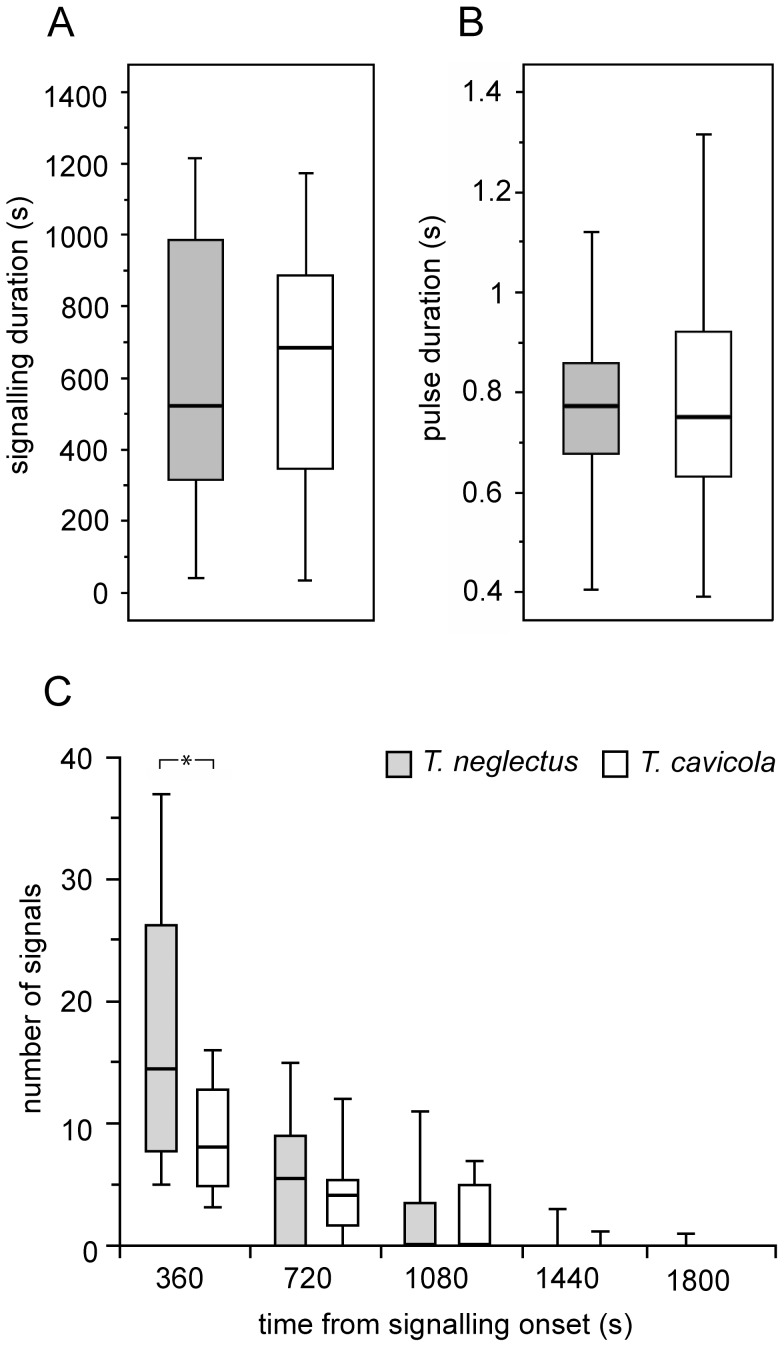
Temporal characteristics of post-copulation signalling of *T. neglectus* and *T. cavicola*. A. Duration of the whole signalling process (T-test; *t*_27_ = 0.79, *P* = 0.937). B. Pulse duration (T-test; *t*_ 135.176_ = −0.748, *P* = 0.456). C. The number of pulses emitted in consecutive 360 s time intervals. In the last two intervals, the left position refers to *T. neglectus*. The asterisk indicates a significant difference in the signal number (T-test values: *t*_25.054_ = 2.716, *P*<0.05). The legend refers to A−C. Data are shown as a box-plot with median and interquartile ranges (outliers excluded).

Shortly after separation from the mate, the males of both species started to shake the whole body vigorously at the place of completed copula (Figure 2, see also below). After slight raising and dorso-ventral bending, the male rocked with intense and clearly visible oscillations. Such behaviour was expressed irrespective of the continued presence of the female which, however, in most cases stayed close to the signalling partner, eating the spermatophore a few centimetres away. Such post-copulation behaviour was performed in *T. neglectus* by all the mated males, while it was absent in two successfully mated males in *T. cavicola* (as well as in those with the unsuccessful SP transfer). The median duration of the whole signalling period was 523 s in *T. neglectus* (Q1−Q3 296.5−1005 s; n = 18) and 684 s in *T. cavicola* (Q1−Q3 207−950 s; n = 11), the difference not being significantly different (Figure 2A; test values in the figure legend). The signals were repeated highly irregularly in both species but, overall, the signal number decreased with time (Figure 2C). The signalling rate was higher in *T. neglectus* than in *T. cavicola*, especially shortly after copulation.

### Vibratory Signals

**Figure 3:**
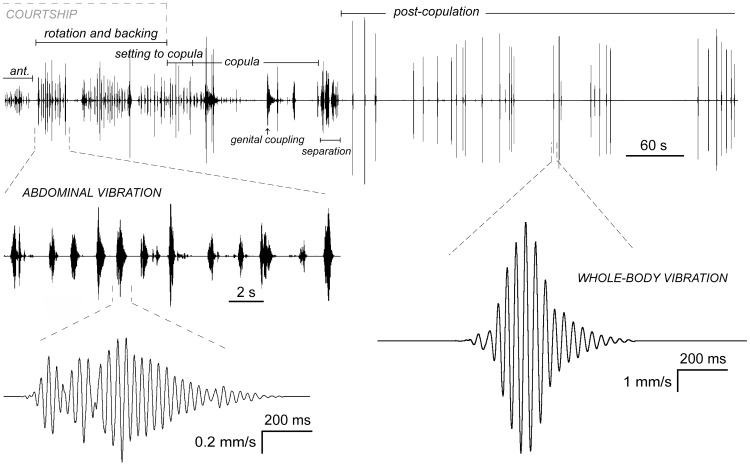
An example of audio recording (oscillograms) from elm bark conducted during and after mating in *T. neglectus*. Enlarged sections show abdominal vibration (left) and whole-body vibration signals (right). Antennation (ant.) and post-copulation phases are shown only partially (as indicated by the open line-end). Just after separation of the mates, recording sensitivity was decreased fivefold.

**Figure 4:**
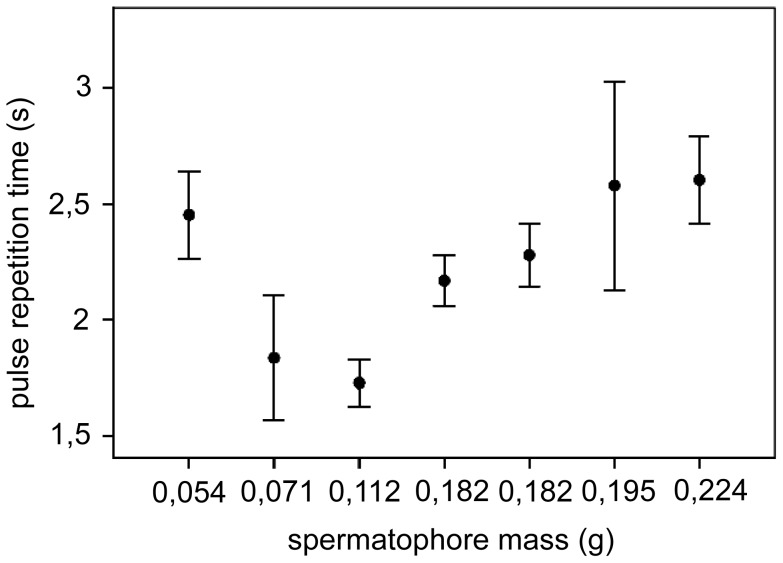
Correlation of the repetition time of courtship pulses with the spermatophore mass in *T. neglectus*. Mean values are shown with the standard error. The signals were recorded for seven of the mated males (n_1_−n_7_ = 23−75), which were weighed to assess the SP mass. Regression test values; *F*_1. 229_ = 47.181, *P*<0.001 for N = 6 (without the first data point)/*F*_1,288_ = 2.951, *P* = 0.087 for N = 7.

**Figure 5:**
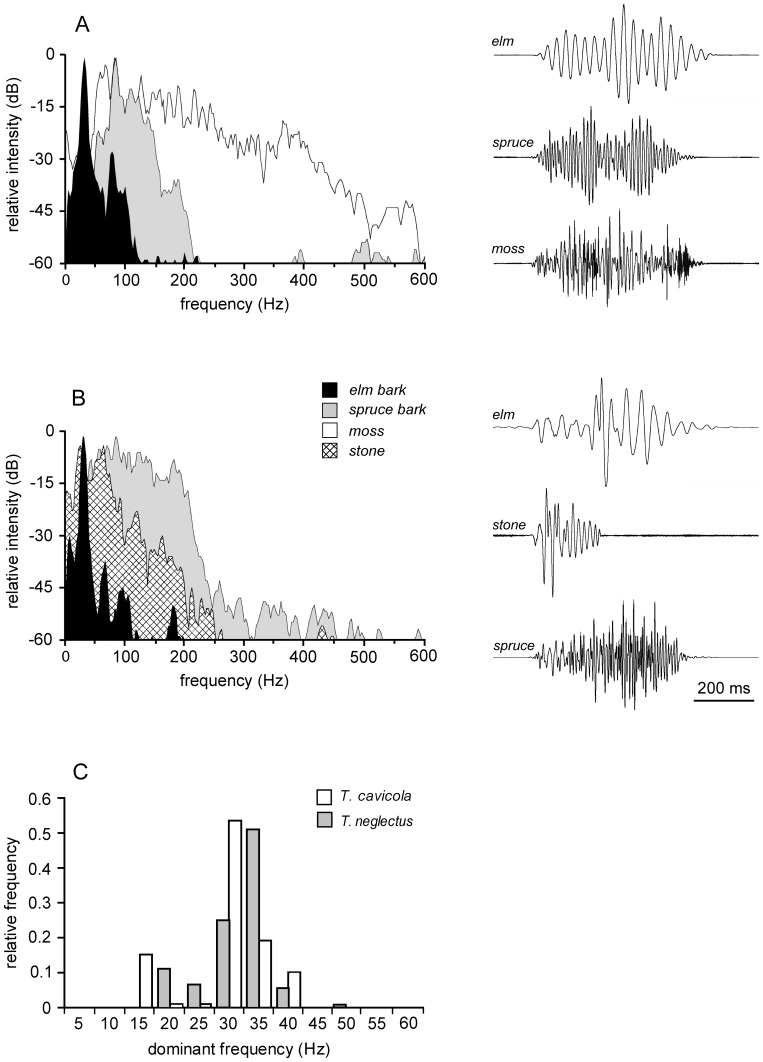
Spectral properties of vibratory signals. Velocity spectra with oscillograms of sample courtship (A) and post-copulation signals (B) of *T. neglectus* recorded on different substrates (the legend and the time scale refers to A and B). C. Distribution of the dominant frequencies in post-copulation signals recorded on elm bark, compared between the two species (n*_TC_* = 99, n*_TN_* = 288).

Courtship signals in *T. neglectus* were produced in sequences of pulses, emitted with a relatively regular repetition time (Figure 3, left). The mean pulse repetition time varied significantly between individuals from 1.728 s (SD 0.253 s) to 2.673 s (SD 0.533 s; ANOVA, *P*<0.001; n_1_−n_14_ = 9−75). Exclusion of the one atypical example resulted in a significant positive regression of the pulse repetition time with respect to the SP mass (Figure 4; test values in the figure legend). The mean pulse duration also varied significantly between individuals, from 0.546 s (SD 0.253 s) to 0.834 s (SD 0.122s; ANOVA, *P*<0.001; n_1_−n_14_ = 12−83). Pulses were amplitude-modulated, typically containing two or three amplitude peaks (Figure 3, left; Figure 5A, right). The mean value of the peak velocity of pulses was 0.184 mm/s (SD 0.149 mm/s) on bark and 0.063 mm/s (SD 0.046 mm/s) on moss. On moss, the mean velocity value was 9.3 dB (dB = 20×log v_1_/v_2_) below the value measured on bark. We recorded no courtship signals above the noise level from the stone.

Post-copulation signalling of both species induced mostly simple, non-amplitude modulated pulses in the substrate (Figure 3, right), while on some locations pulses contained two main amplitude peaks (Figure 5B, right). The mean pulse duration on bark varied between individuals from 0.590 s (SD 0.098 s) to 1.024 s (SD 0.276; n_1_−n_11_ = 7−57) in *T. neglectus* and between 0.560 s (SD 198 s) and 1.105 s (SD 0.184 s; n_1_−n_8_ = 4−34) in *T. cavicola*, with no significant differences among the species (Figure 2B; test values in the figure legend). The mean value of the peak velocity of pulses was on bark 3.556 mm/s (SD 1.621 mm/s) in *T. neglectus* and, similarly, 2.161 mm/s (SD 1.78 mm/s) in *T. cavicola*. In *T. neglectus* the mean velocity of the post-copulation signals was 25.7 dB above the velocity of courtship signals on bark. On the stone the mean pulse duration, as recorded for *T. neglectus*, was 0.256 s (SD 0.024 s; n = 10) and the mean value of the peak velocity was 0.135 mm/s (SD 0.04 mm/s). The mean signal velocity on the stone was 28.4 dB below the value on bark.

Signals’ spectral properties were substrate-dependent. On the same substrate the frequency spectra were similar in both species for both signal types (Figure 5; Table 1). The spectrum was extremely narrowly banded on elm bark, with most of the energy content in the range below 150 Hz. On spruce bark and, similarly, on the stone, most spectral energy was emitted below 250−300 Hz, while on moss the frequency spectrum extended up to 600 Hz, with prominent peaks expressed in the range up to 400 Hz. The dominant frequency was expressed below 120 Hz on all substrate types.

**Table 1:** Dominant frequencies of signals recorded on different substrates.

Species	Signal type	Dominant frequency (Hz)			
		elm bark	spruce bark	moss	stone
*T. neglectus*	C	31/28–32 (173/8)	82/76−88 (156/5)	82/72−186 (20/1)	
	PC	31/30−32 (288/10)	111/88−117 (23/1)		41/40−46 (10/1)
*T. cavicola*	PC	27/26−31 (99/8)			

C: courtship signals (not emitted by *T. cavicola*), PC: post-copulation signals.

Median values and interquartile ranges are shown with the number of recorded signals and males (n/N).

## Discussion

Rhaphidophoridae are very difficult to observe under natural conditions, due to their nocturnal activity, negative phototaxis and sojourn mainly in underground habitats. This appears to be the main reason for the scarceness of data on their mating behaviour, including the lack of descriptions of vibratory signalling so far.

In the strongly seasonal life cycles of the two investigated species, the mating periods are separated by at least two months [45], [46], which appears to constitute an effective reproductive barrier between them. This suggests the absence of a strong sexual selection for diversification of their behaviour, otherwise typical for the closely related species in the secondary contact [52], [53]. The significant differences observed in their mating behaviour may thus largely reflect environmental adaptations. As will be discussed in more detail below, such behavioural adaptations may be presumed for *T. cavicola*, while the behaviour of *T. neglectus* may be considered ancestral.

### Signal Transmission, Detection and Environmental Constraints

We showed that both modes of signal production in the investigated species − vibration of the whole body and of the abdomen − induce narrow-band, low-frequency vibrations in the substrate. Vibrational signals, with most of the spectral energy below a few hundred Hz and the dominant peak between 10−100 Hz, are also produced by tremulation in other Ensifera [13], [15], [54]. In general, such signals are well suited for transmission in various types of natural substrates, such as plants [55], [56], [57], wood [58], soil [59], [60], sand and rock [61], which all act as low-pass filters for transmitted vibrations. Rock, however, with its extremely high mechanical impedance, is the least appropriate for vibrational communication [61].

To assess the efficiency of the cave crickets’ signalling on different substrates, we compared the spectral and intensity characteristics of their signals with the tuning of the central vibratory neuron network investigated in *T. neglectus* [62]. The majority of ventral nerve chord vibratory neurons in the latter species respond most sensitively to vibrations below 400 Hz, with the lowest thresholds at 0.005–0.02 m/s^2^. The mean velocity of the courtship signals in *T. neglectus* was on bark at a dominant frequency around 14 dB above, and on moss around 17 dB above, the sensory threshold of the (adjacent) female receiver. The velocity of stronger post-copulation signals was on bark about 36−40 dB above, and on rock around 10 dB above, the interneuron threshold (which may be presumed similar for both species). This intensity difference between bark and rock suggests that, on rock, *T. neglectus* courtship signals, which were undetectable by the laser vibrometer, were emitted at intensities below the species’ physiological range of detection. Insufficient signal detection may thus be part of the reason why, in the only case of *T. neglectus* mating on the stone, the male courted the female much longer than all other successfully mated males.

Mating in both investigated species took place most often on bark, which may have been selected for its inclined position in the set-ups. On the inclined substrates, the ventilatory activity of cave crickets is decreased and they are less sensitive to external stimuli [63], [64], which may be preferred for pair formation. For *T. neglectus* these mating sites may also indicate substrate selection. Despite the lack of systematic field observations, mating of this species − which occurs months after they leave overwintering places − is supposed to take place predominantly outside caves (T. Novak, personal communication). As bark and hollow trunks are often inhabited by the cave crickets during the summer [43], they may constitute a frequent substrate for mating and signalling in *T. neglectus*, which, in our study, enabled emission of their signals at the highest intensity. Also moss, as measured in our study, and litter on the forest ground would enable them effective vibratory communication [55], [61]. In *T. cavicola*, on the other hand, maturation and mating starts immediately after the end of their winter diapause [45]. At this time copulations were several times encountered on cave walls ([42], T. Novak, personal comm.) and females with spermatophores have also been found in deep cave parts (our own observations; T. Novak, personal comm.), indicating that mating in this species takes place at least in part before individuals migrate outside.

The specific mating habitats of the investigated species could largely explain the differences in their mating strategies. The communication medium is generally regarded to strongly influence the evolution of signals and the signalling behaviour [65], [66]. These effects are especially strong in vibratory communication, where different signalling substrates may be a primary cause of species divergence [67]. It may be presumed that the high attenuation of vibratory signals transmitted through rock, as demonstrated by our study, was responsible for the reduced abdominal vibration signalling in courtship of *T. cavicola*. The prolonged antennation phase in this species, in which the females are also more active overall compared to *T. neglectus*, appears to compensate for the absence of vibratory information during courtship. The prolonged copulation phase in *T. cavicola*, on the other hand, does not appear to have any specific function (see [10], [68]) and may simply reflect the mating of this species in caves in the absence of natural enemies.

In addition, differences in pre-mating behaviour between the species suggest different strategies for mate location (Stritih in prep.), which may also be ecologically founded. The absence of signalling by scent gland protrusion and generally lower degree of locomotory activity in males of *T. cavicola* than in *T. neglectus* indicate that other factors, like their gregarious distribution in caves [69], facilitate pair formation.

The courtship behaviour of *T. neglectus* is similar to that described for other Rhaphidophoridae such as the epigean *Tachycines asynamorus* [37] and the troglophilic *Ceuthophilus guttulosus* [41]. Body stroking of females in the former, and abdominal “twitching” in males approaching females in the latter, may indicate the presence of vibratory signalling. No such behavioural indications exist, on the other hand, for troglobionts [30], [38], [40]. We may therefore hypothesise that signalling in courtship, such as expressed by *T. neglectus*, is a primitive trait in this family and has been lost in the course of adaptation to cavernicolous life. To confirm this, vibratory signalling needs to be investigated in further species of Rhaphidophoridae that live in various habitats.

### Signalling Function

In *T. neglectus*, courtship signalling may be advertising the male’s presence, decreasing female sensitivity to disturbing stimuli [70], and/or providing her with information on the male’s quality [71], [72]. The unsuccessful courting observed for several males and, especially, a case of female rejection of the first male while accepting its rival shortly after (data not shown), indicate that the females of this species can be choosy. The high inter-individual variability of both temporal parameters measured in the courtship signals, and especially the positive correlation observed between the pulse repetition time and the spermatophore mass, may have served as a basis for the female choice. A similar feature, although with a reverse correlation, was shown in the katydid *Conocephalus nigropleurum* [15]. In this species the females prefer vibratory signals with a shorter repetition time, which is indicative of a larger male − and thus of a larger spermatophore with spermatophylax (see below).

The role of post-copulation signals emitted by the two *Troglophilus* species is less clear than the role of the courtship signals. In crickets, auditory and/or vibratory displays occur after copulation to prevent female remating with other males and/or premature removal of the sperm ampula [2], [10], [11]. In katydids such behaviour is largely absent, since the sperm is protected by the spermatophylax, which the female consumes after copulation [3], [73]. As both species investigated here provide the female with a large spermatophylax, a “guarding” function of their post-copulation signalling is unlikely. Indeed, such behaviour is expressed irrespective of the presence of the mated female. Nevertheless, an effect on the female is suggested by the highest frequency of signals emitted in the first minutes after copulation, when the chance of her being in the vicinity is greatest. Also, these high-intensity signals may operate over relatively long distances. We may speculate that such signalling has a physiological effect on the females, which increases the male fitness, e.g. by increasing fertilisation success. The lower signalling rate in *T. cavicola* than in *T. neglectus*, together with the absence of such behaviour in some mated males, may again suggest evolutionary regression due to the low effectiveness of signal transmission in caves.

### The Place of Rhaphidophoridae in Signalling Modes of Ensifera

Signalling by vibration or tremulation of the body or of some of its parts, is one of the most widespread and, presumably, primitive modes of mechanical signalling in insects [74], [75]. In Ensifera, such signalling occurs during the close-range courtship in most cricket and many katydid species [10], [11], [13], [14], [15], following the attraction of the female by the airborne sound signals of the male. The more complex signalling by tremulation, on the other hand, which functions, in addition, in mate calling and may include female replies, has been found among the katydid species, together with reduced sound communication [54]. Tremulation is also part of the courtship display in various cockroaches, including the basal lineages [24], [76], [77]. These data suggest that courtship signalling by tremulation can be considered primitive for Ensifera, probably being inherited directly from a cockroach-like ancestor. The tremulation now described as part of courtship in *T. neglectus* is in line with the proposed primitive position of Rhaphidophoridae within Ensifera [23], [27], [28] and in turn provides an additional argument in its support.

In other non-hearing families of Ensifera, the mechanosensory communication is much more elaborate than in the cave crickets, since it includes not only long-distance mate calling but also male-female duetting via drumming vibrations, along with their ability to emit stridulatory sound [6], [7]. Correspondingly, the construction of the vibratory tibial organs of these species is much more complex than in Rhaphidophoridae [78], since they possess an additional sensory part homologous to the “crista acoustica” of the ensiferan ear [25], [28], [29]. This presumed precursor organ for audition [28] has been proposed to have evolved for enhanced detection of intraspecific vibratory signals, potentially focusing on their high frequency components [25]. Not directly supporting, but nevertheless not contradicting this hypothesis, is the low-frequency nature of the cave cricket’s signals produced by tremulation. In the course of evolution, such signals apparently did not provide the required sensory drive for a functional extension of the tibial organ in Ensifera. This appears to have been associated with other signalling modes, such as are drumming and/or stridulation, which induce substrate vibration (and sound) with frequencies extending up to several kHz [6], [8], [13].

In conclusion, our paper provides the so far missing behavioural characters for Rhaphidophoridae, which are in line with the proposed primitive position of the group within Ensifera. The behaviour, and vibratory signals, described for two closely related species with different life habits, enabled inferences to be made as to the mechanosensory evolution of Ensifera at different scales.

## Materials and Methods

### Animals and Experimental Conditions

Experiments were carried out on mature *T. neglectus* and *T. cavicola* in 2009 and 2011, within two mating seasons of the former and one of the latter species. Subadult and last larval instars of *T. neglectus* were collected between mid June and July in two caves near Tolmin (north-western Slovenia). Subadult *T. cavicola* were collected in mid February in a cave near Žalec (north-eastern Slovenia). The animals were kept in the laboratory separated by sex, at 20−22°C and relative humidity 50−70%, under a light/dark photo cycle of 16/8 h, for 2−6 weeks before experiments. They were fed *ad libitum* with aquarium fish food.

Experiments were conducted in a series of 3−5 glass terrariums (24×24×38 cm) filled with a layer of water-soaked turf covered by a layer of moss, and furnished with a piece of limestone (ca. 5×7−10×5−7 cm) and 2 pieces of bark (ca. 5−10×20−25 cm) positioned inclined against the terrarium wall. Spruce bark was used in the first season with *T. neglectus* and elm bark in the others. Small pieces of reflecting tape (ca. 5×5 mm), for better reflection of the laser beam, were attached to the surfaces of bark and stones, with no more than 3 cm distance between them. As moss was inappropriate to be fully covered with pieces of reflective tape, we attached one piece to its surface, adjacent to a mating pair in one experiment. Terraria had a metal-net cover with openings of ca. 5 mm to enable passage of the laser beam. The laboratory was ventilated to prevent excessive concentration of odours.

Two males and three females, marked individually by different combinations of points on the thorax, were set per terrarium and three to five such terraria were observed at the same time. Animals were observed under red illumination (λ >610 nm; undetectable by these species [51]) continuously through 5−8 hours of the dark phase, between 16^th^ March and 24^th^ April 2011 for *T. cavicola*, and between 6^th^ August and 4^th^ September 2009 and again between 27^th^ July and 19^th^ August 2011 for *T. neglectus*. During the light phase and weekends, males were separated from females and returned to them 15 min prior to the onset of observation. To determine spermatophore mass, a male was weighed before and after mating, when the mated pair was replaced by a new pair.

### Data Acquisition and Analysis

Every 15 minutes the context of activity of each individual was registered, its position in the terrarium, and the state of protrusion of scent glands in males, on hand-written notes following ca. one minute of observation per terrarium. In addition, their behaviour was recorded using two camcorders (Canon XM2) and, simultaneously, their vibratory emissions using a laser vibrometer (OFV 505 Sensor head, OFV 5000 Controller; Polytec GmbH, Waldbronn, Germany) at distances of up to 5 cm from the signalling males. Courtship signals of *T. neglectus* were recorded for thirteen males from bark (elm: N = 8, n = 173; spruce: N = 5, n = 158; where N is the number of males and n the total number of signals), for one from moss (n = 20), and for two from the terrarium net cover (n = 89; but these recordings were not considered for the analysis, since the signals were distorted in the time and frequency domains relative to those on the natural substrates). Post-copulation signals were recorded in both species from bark (elm: N = 10, n = 288/spruce: N = 1, n = 24 in *T. neglectus*; elm: N = 8, n = 99 in *T. cavicola*) and in one *T. neglectus* male from the stone (n = 10). Vibration records were stored on a computer via a sound card (24-bit, 192 kHz; Sound blaster X-Fi Notebook, Creative Labs Inc., Milpitas, CA) using Cool Edit Pro software (2.0, Adobe Systems Inc., San Jose, CA) at a sample rate of 44100 Hz and with 16 bit resolution.

In the cases where several successive male courtship attempts were needed to induce the female’s response, temporal analysis of the mating process was conducted only for the last sequence leading to copulation. The time from the onset of the female’s response (climbing the male’s back) until the mates were set in the typical copulatory position was considered as the phase of establishing the copula.

Temporal, spectral and intensity parameters of vibratory signals were measured in Sound Forge 6.0 (Sonic Foundry Inc., Madison, WI). Pulses refer to homogenous parcels of vibrations of finite duration, and pulse repetition times to the periods between the onsets of two consecutive pulses. Frequency analysis was based on the FFT size of 32.768 smpl, and the display range of 60 dB (dB = 20×log v_1_/v_2_) normalised to the dominant peak amplitude. Pulse intensity refers to the positive peak velocity for the cycle with the largest amplitude. In the absence of a vibration record, the duration of post-copulation signalling and the number of emitted pulses was determined from video records.

For further analysis and statistical evaluation of the behavioural data and the signals’ parameters we used KyPlot 5.0 (KyensLab Inc., Tokyo, Japan) and SPSS 13.0 (IBM, USA). Intraspecific variation was evaluated by ANOVA, and interspecific differences by the Student’s T-test.
